# Protecting the regenerative environment: selecting the optimal delivery vehicle for cartilage repair—a narrative review

**DOI:** 10.3389/fbioe.2024.1283752

**Published:** 2024-01-25

**Authors:** T. Mark Campbell, Guy Trudel

**Affiliations:** ^1^ Elisabeth Bruyère Hospital, Ottawa, ON, Canada; ^2^ Bone and Joint Research Laboratory, Ottawa Hospital Research Institute, Ottawa, ON, Canada; ^3^ Department of Cellular and Molecular Medicine, Faculty of Medicine, University of Ottawa, Ottawa, ON, Canada; ^4^ The Ottawa Hospital, Department of Medicine, Division of Physical Medicine and Rehabilitation, Ottawa, ON, Canada

**Keywords:** osteoarthritis, cartilage, stem cells, musculoskeletal health, regenerative therapy

## Abstract

Focal cartilage defects are common in youth and older adults, cause significant morbidity and constitute a major risk factor for developing osteoarthritis (OA). OA is the most common musculoskeletal (MSK) disease worldwide, resulting in pain, stiffness, loss of function, and is currently irreversible. Research into the optimal regenerative approach and methods in the setting of either focal cartilage defects and/or OA holds to the ideal of resolving both diseases. The two fundamentals required for cartilage regenerative treatment are 1) the biological element contributing to the regeneration (e.g., direct application of stem cells, or of an exogenous secretome), and 2) the vehicle by which the biological element is suspended and delivered. The vehicle provides support to the regenerative process by providing a protective environment, a structure that allows cell adherence and migration, and a source of growth and regenerative factors that can activate and sustain regeneration. Models of cartilage diseases include osteochondral defect (OCD) (which usually involve one focal lesion), or OA (which involves a more diffuse articular cartilage loss). Given the differing nature of these models, the optimal regenerative strategy to treat different cartilage diseases may not be universal. This could potentially impact the translatability of a successful approach in one condition to that of the other. An analogy would be the repair of a pothole (OCD) *versus* repaving the entire road (OA). In this narrative review, we explore the existing literature evaluating cartilage regeneration approaches for OCD and OA in animal then in human studies and the vehicles used for each of these two conditions. We then highlight strengths and challenges faced by the different approaches presented and discuss what might constitute the optimal cartilage regenerative delivery vehicle for clinical cartilage regeneration.

## 1 Introduction

Focal cartilage defects are common and cause significant morbidity, including pain, swelling, mechanical symptoms (such as a feeling of joint instability), and functional disability (Mandelbaum et al., 1998; Liu et al., 2022). Over a quarter of asymptomatic middle-aged individuals with no family history of cartilage loss were found to have some degree of focal cartilage defect; those with a family history of knee osteoarthritis (OA) demonstrated a cartilage defect prevalence of >50% (Ding et al., 2007; Everhart et al., 2019). In those with symptomatic knee OA, cartilage defects have a prevalence of >80% (Ding et al., 2007; Everhart et al., 2019). Correspondingly, cartilage defects represent an underrecognized, yet major risk factor for the development of OA (Mandelbaum et al., 1998; Arøen et al., 2004; Cicuttini et al., 2005; Ding et al., 2007; Guermazi et al., 2017; Everhart et al., 2019; Liu et al., 2022). While loss of the articular cartilage is often described as the hallmark of OA, OA is a total joint disease also affecting bone, synovium, meniscus and other intra- and extra-articular tissues such as ligament and muscle (Barr et al., 2015; Martel-Pelletier et al., 2016; Cunha et al., 2019; Hunter and Bierma-Zeinstra, 2019). Pain, loss of function, joint stiffness and reduced quality of life are well-described sequelae of OA (Barr et al., 2015; Martel-Pelletier et al., 2016; Hunter and Bierma-Zeinstra, 2019; Campbell and McGonagle, 2020). Additionally, while cartilage defects are a risk factor for OA (Martel-Pelletier et al., 2016; Hunter and Bierma-Zeinstra, 2019), OA can occur independently of a pre-established focal cartilage defect (Ding et al., 2007; Everhart et al., 2019). In addition, detection of focal cartilage defects requires advanced imaging (e.g., MRI), and may not be appreciated until more advanced stages. As such, patients will often present for medical treatment at more advanced stages of cartilage degeneration and joint dysfunction, with OA already established (Finley et al., 2018). The optimal method of regenerating cartilage for both focal cartilage defects and OA is actively researched (Clinicaltrials.gov, 2023) with the hope of discovering a treatment that could resolve both pathologies.

Stem cells have been delivered to cartilage lesions (both focal cartilage defects and OA) in the clinical setting before basic or translational evidence supported the regenerative efficacy of the various approaches (Marks et al., 2017; Canada, 2019; ISfSC, 2021). Stem cell delivery was rapidly extended to clinical practice, often at high cost to the patients (Marks et al., 2017; Canada, 2019; ISfSC, 2021). These two factors led national healthcare governing bodies to step in and limit this practice (Marks et al., 2017; Canada, 2019; ISfSC, 2021). The upside was an expansion of the basic science and translational research in this area to build a more solid foundation upon which to eventually base clinical regenerative treatment. Two major components of this regenerative treatment include 1) the biological element contributing to the regeneration (e.g., stem cells, extravesicular vesicles), and 2) the vehicle by which these biological elements are delivered (e.g., organic fluids, engineered scaffolds) (Campbell et al., 2022). Proposed advantages of using stem cells or EVs as the biological element have been reviewed previously (Kim et al., 2020; Taghiyar et al., 2021; Campbell et al., 2022) and include a direct ability to repair damaged cartilage (for stem cells), immunomodulation capacity to reduce inflammation within the OA joint, as well as paracrine effects that recruit other endogenous regenerative cells to the site of injury. While these potential advantages have been described for stem cells and EVs, there is currently no consensus regarding which method is more advantageous. The vehicle should be considered equally important to the biological element as it supports the regenerative process. A protective 3-dimensional (3D) environment may preserve stem cells’ ability to differentiate; a structure may allow cell adherence and migration; a source of growth and regenerative factors may activate and sustain regeneration (Kim et al., 2019).

Various animal models of cartilage injury have been described, such as: focal osteochondral defects (OCD; a localized osteochondral defect surgically imposed through the articular surface) and various OA models such as the destabilization of the medial meniscus (DMM) model, the anterior cruciate ligament transection (ACLT) model, and chemical models for generalized OA-like cartilage loss; these models are described in detail by Cully (2015) (Culley et al., 2015) and Cope (2019) (Cope et al., 2019). OCD *versus* OA models cause different sizes and distributions of cartilage injuries, and potentially differing endogenous intra-articular responses. Consequently, the model may impact the optimal regenerative delivery vehicle selected to the specific injury or condition in need of repair when moving from translational to clinical therapy (Liu et al., 2022).

In this narrative review, we evaluated the existing literature on cartilage regeneration both focal OCDs and OA in both animal models and clinical trials. Since the optimal stem cell population (Campbell et al., 2022), or use of their secretome (Kim et al., 2020; Taghiyar et al., 2021), for cartilage regeneration has been reviewed elsewhere, we focus here on the vehicles used in these studies and consider both the context in which they were used, as well as their general applicability to focal OCDs and OA. We conclude by looking towards the application of scaffold technology in clinical trials.

## 2 Animal models of focal osteochondral defects

The last decade saw advancements in vehicle engineering to treat focal OCDs in animal models (Lam et al., 2014; Shimomura et al., 2014; Li et al., 2015a; Dashtdar et al., 2015; Ha et al., 2015; Goodrich et al., 2016; Park et al., 2017; Ruan et al., 2017; Li et al., 2018; Lu et al., 2018; Muttigi et al., 2018; Ye et al., 2018; Jia et al., 2019; Jin et al., 2019; Kondo et al., 2019; Park et al., 2019; Pascual-Garrido et al., 2019; Wang et al., 2019; Xu et al., 2019; Cho et al., 2020; Wu et al., 2020; You et al., 2020; Deng et al., 2021; Heirani-Tabasi et al., 2021; Murata et al., 2022; Zheng et al., 2022) (Table 1). In the rat, intra-articular needle injection delivered the vehicle, such as hyaluronic acid (HA) hydrogels (Li et al., 2018; Xu et al., 2019). While direct placement of the vehicle into the OCD, a more precise delivery of the vehicle is limited by the technical challenges of placing a pre-formed scaffold in a small lesion (∼1.5–2 mm in width and <1 mm in depth in rats, smaller in mice). Two studies reported improved histologic-based cartilage scoring outcomes after implanting a gel or cell pellet scaffold into the OCD of the rat knee (Xu et al., 2019; Zheng et al., 2022). The most common model however remains the rabbit knee OCD (Table 1); providing a relatively large joint for surgical scaffold implantation handled with the naked eye and relatively lower cost (*versus* dog or horse, for example,). In the mid-2010s, vehicles used in rabbit OCD models included bilayered hydrogels that cross-linked at body temperature (Lam et al., 2014), synthetic hydroxyapatite (Shimomura et al., 2014) and Polyvinyl alcohol-chitosan composite hydrogel (Dashtdar et al., 2015), implanted during open knee surgery. Nanofiber scaffolds, composed of ultra-fine biodegradable polymeric fibers that simulate the natural extracellular matrix (ECM), have also emerged in animal OCD studies (Kazemnejad et al., 2017; Ahmadian et al., 2023). Nanofiber scaffolds may be constructed using different techniques, including electrospinning, self-assembly, phase separation, and drawing (Ahmadian et al., 2023). Natural nanofiber hydrogel scaffolds (scaffolds with high water content and elastic properties with cross-linked, multiporous networks (Li et al., 2019)) include alginate, gelatin, agarose, HA, fibrin, and collagen, while synthetic scaffolds often contain polycaprolactone, polyethylene glycol, polyurethane, poly (pdioxanone), and/or poly (lactic acid) (Kazemnejad et al., 2017; Ahmadian et al., 2023). For the interested reader, reviews describing the advantages of nanofiber scaffold technology are available (Coburn et al., 2012; Kazemnejad et al., 2017; Ahmadian et al., 2023).

**TABLE 1:** Small and large animal osteochondral defect studies and associated injection vehicle.

Author (Year)	Animal (n)	Joint	Cell type	Vehicle	Implantation method
Lam et al. (2014)	Rabbit (24)	Knee	BM-MSCs	Bilayered HA hydrogel with 37°C cross-linking	Implantation during open surgery
Shimomura et al. (2014)	Rabbit (41)	Knee	SD-MSCs	3D cellular tissue construct and NEOBONE^®^ synthetic hydroxyapatite	Implantation during open surgery
Dashtdar et al. (2015)	Rabbit (24)	Knee	BM-MSCs	Polyvinyl alcohol-chitosan composite hydrogel	Implantation during open surgery
Ha et al. (2015)	Minipig (6)	Knee	UC-MSCs	HA hydrogel	Implantation during open surgery
Li et al. (2015a)	Rabbit (30–60 knees)	Knee	BM-MSCs	bi-layered scaffold of poly vinyl alcohol, collagen-derived gelatin, vanillin, nano-hydroxyapatite/polyamide-6	Implantation during open surgery
Goodrich et al. (2016)	Horse (12)	Knee	BM-MSCs	Platelet-enhanced fibrin	Injection during open surgery
Park et al. (2017)	Rabbit (40)	Knee	UC-MSCs	HA hydrogel	Implantation during open surgery
Ruan et al. (2017)	Rabbit (16)	Knee	BM-MSCs	Biphasic silk-fibroin/chitosan and silk-fibroin/chitosan/nano-hydroxyapatite scaffold	Implantation during open surgery
Ye et al. (2018)	Rabbit (12)	Knee	AT-MSCs	Rabbit-derived acellular dermal matrix	Implantation during open surgery
Li et al. (2018)	Rat (30)	Knee	Human intra-articular flushed fluid MSCs	Cross-linked hyper-branched poly (ethylene glycol) diacrylate/HA hydrogel	Injection during open surgery
Lu et al. (2018)	Rabbit (24–48 knees)	Knee	Endogenously-recruited BM-MSCs	Cartilage acellular matrix scaffold combined with a bone marrow stem cell-homing self-assembling peptide creating a 3D hydrogel	Implantation during open surgery
Muttigi et al. (2018)	Rats (36)	Knee	AT-MSCs	Matrilin-3 in HA	Implantation during open surgery
Jia et al. (2019)	Rabbit (18)	Knee	SD-MSCs	Glycol chitosan and benzaldehyde capped poly (ethylene oxide) hydrogel	Injection during open surgery
Jin et al. (2019)	Rabbits (12)	Knee	BM-MSCs	Poly (L-lactide) and gelatin fibrous meshes	Implantation during open surgery
Kondo et al., (2019)	Microminipig (13)	Knee	SD-MSCs	Hanging drop MSC aggregates	Implantation during open surgery
Park et al. (2019)	Rat (20)	Knee	UC-MSCs	HA hydrogel	Implantation during open surgery
Pascual-Garrido et al. (2019)	Rabbit (10)	Knee	BM-MSCs	Photopolymerizable multi-component (PEG, ChS, peptide crosslinker, RGD), cartilage-mimetic hydrogel	Injected during open surgery then cross-linked under blue light
Wang et al. (2019)	Rabbit (22)	Knee	Endogenously-recruited BM-MSCs	Aptamer-modified silk fibroin sponge embedded into silk fibroin/hyaluronic acid–tyramine hydrogel	Implantation during open surgery
Xu et al. (2019)	Rat (50)	Knee	BM-MSCs	Gelatin supramolecular “Host-Guest Macromer” hydrogel (HGM) or chemically crosslinked methacrylated gelatin hydrogel (GelMA)	Either injection (HGM) or press fit (GelMA) into defect
Cho et al. (2020)	Rabbit (40)	Knee	AT-MSCs	Poly (ethylene argininylaspartate diglyceride) polycation, heparin, and insulin-like growth factor-1 in thiolated gelatin (gelatin- SH)/poly (ethylene glycol) diacrylate interpenetrating network hydrogels	Implantation during open surgery
Wu et al. (2020)	Rabbit (24)	Knee	BM-MSCs	2D-nanopattern differentiated cells delivered as a bilayered construct including fibrin in upper layer	Sequential injection of each layer into defect during open surgery
You et al. (2020)	Rabbit (20)	Knee	AF-MSCs	Fibronectin and binding protein sheets (cell-secreted)	Implantation during open surgery
Deng et al. (2021)	Rabbit (40)	Knee	BM-MSCs	Printed parathyroid hormone-coupled silk fibroin and gelatin methacryloyl scaffold with biomechanical gradient	Implantation during open surgery
Heirani-Tabasi et al. (2021)	Rabbit (12–24 knees)	Knee	AT-MSCs	Chitosan and HA	Implantation during open surgery
Murata et al. (2022)	Horse (3)	Knee	SD-MSCs	3D cell-based spheroids in a cylindrical mold	Implantation during open surgery
Zheng et al. (2022)	Rat (16)	Knee	Wharton’s jelly MSCs	As a cell pellet	Implantation during open surgery

AF, amniotic fluid; AT, adipose tissue-derived; BM, bone marrow derived; ChS, chondroitin sulfate; HA, hyaluronic acid; MSCs, mesenchymal stromal cells; PEG, polyethylene glycol; RGD, arginyl-glycyl-aspartic acid; SD, synovium-derived; UC, umbilical cord.

Since the evolution of nanofiber technology, more sophisticated vehicles have been bioprinted that incorporated distinct osteogenic and chondrogenic regions or zones matching the anatomy of the predefined OCD (Yilmaz et al., 2020). For example, 2D-nanopattern chondroitin sulfate-coated scaffolds featured an inverse pattern of nanograting (250-nm line, 250-nm space, and 150-nm height) or nanohole (225-nm diameter, 400-nm pitch, and 300-nm height) patterning with cells delivered as a bilayered construct (Wu et al., 2020). In doing so, it was hypothesized that stem cells differentiated on specific nanotopographic patterns based on a zonally stratified cartilage construct would possess topographically induced mechanical memory, and could thus retain an induced chondrogenic phenotype (Wu et al., 2020). Correspondingly, these constructs included layer-defined pre-differentiation of incorporated mesenchymal stem cells (MSCs) to enhance their osteogenic and/or chondrogenic abilities, depending on where they were implanted (subchondral bone or articular surface) (Wu et al., 2020). In addition to stem cells, various growth factors have also been incorporated into these scaffolds to enhance anabolic cellular activity. For example, a biphasic scaffold utilizing gelatin methacryloyl (GM) in one phase and parathyroid hormone (PTH) covalently immobilized on silk fibroin (SF) with methacrylic anhydride (MA) in a second resulted in a corresponding biomechanical gradient due to the differing physical properties of the constituents (Deng et al., 2021). Benefits of potential growth factor use, such as insulin-like growth factor, fibroblast growth factors, bone morphogenic proteins and transforming growth factor beta, for cartilage regeneration have recently been reviewed (Hernigou et al., 2022; Trippel, 2023); however a growth factor that robustly augments articular cartilage healing remains elusive (Trippel, 2023).

Another interesting approach to OCD repair was injecting a mimetic hydrogel composed of polyethylene glycol, chondroitin sulfate, a matrix metalloprotein-2 degradable peptide crosslinker, and arginyl-glycyl-aspartic acid (Pascual-Garrido et al., 2019). This scaffold allowed cells encapsulation during the hydrogel formation process, with the final combination being formed *in situ* within the articular chondral defect, then cross-linked to maintain shape (Pascual-Garrido et al., 2019). In this study, the authors included the matrix metalloprotein-2 degradable peptide cross-linking photoinitiator (CVPLSLYSGC) that polymerized under blue light. After the defect was generated, a red light in the operating room allowed defect visualization and polymer delivery. Polymer solution was then injected onto the chondral defect, then photopolymerization was performed *in situ* by use of a 405-nm blue light in order to establish the final placing of the customized regenerative material (Pascual-Garrido et al., 2019). The majority of small animal OCD studies reported positive structural outcomes following stem cells delivery.

Larger animals studies of OCD have included the use of HA hydrogel (Ha et al., 2015) and hanging drop MSC aggregates (Kondo et al., 2019) in minipigs, as well as platelet-enhanced fibrin (Goodrich et al., 2016) and 3D-based microspheroids (Murata et al., 2022) in horses. These proof-of-concept studies (Table 1) often reported positive structural outcomes following stem cell application.

**FIGURE 1:**
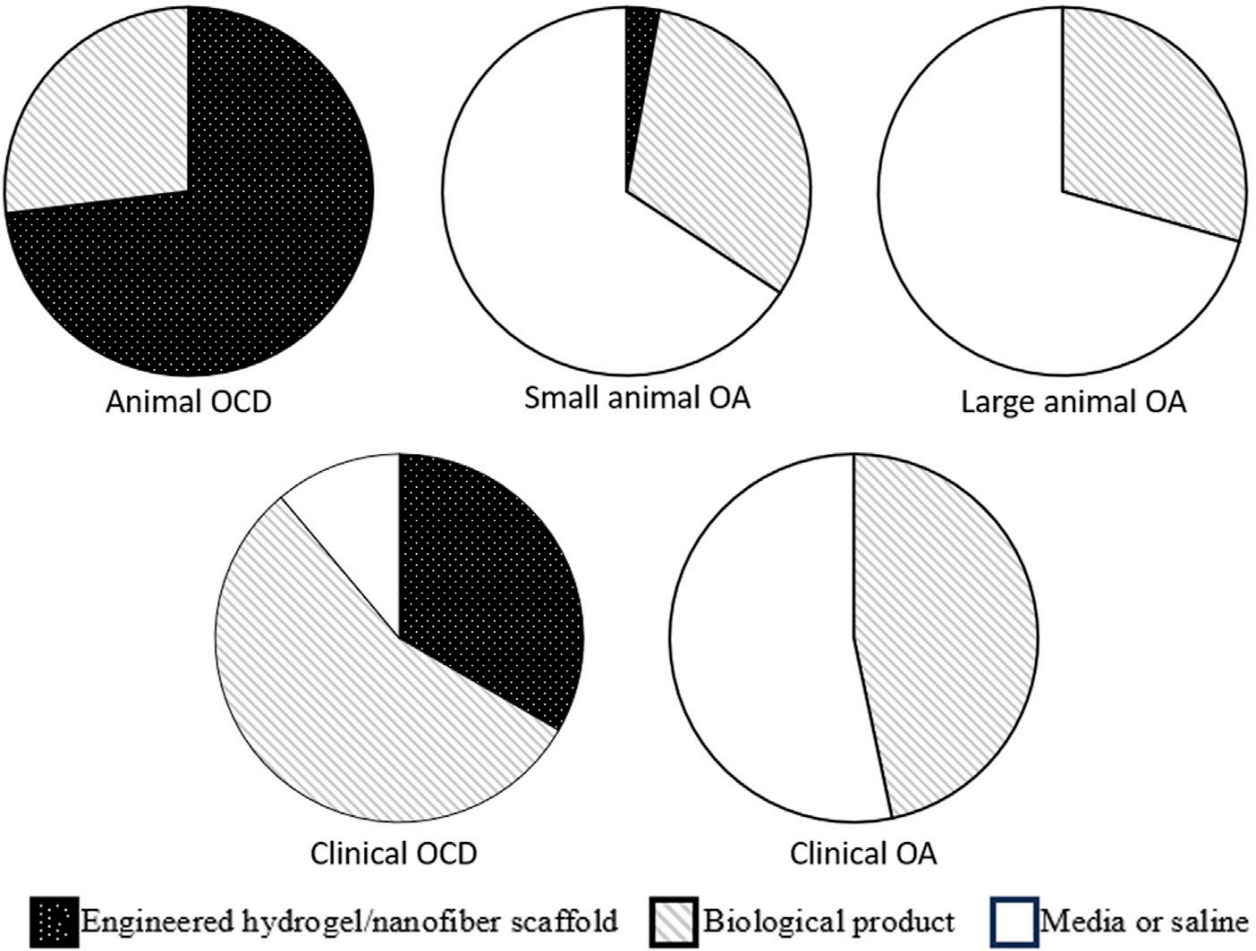
Graphical summary of scaffold types used in animal and clinical models of osteochondral defect and osteoarthritis. It can be seen that the proportion of engineered scaffolds is much larger in OCD models (animal models in particular), as compared to OA. OCD, osteochondral defect; OA, osteochondral defect.

Overall, scaffolds used over the last decade evaluating the treatment of OCDs in animal models have been roughly evenly divided between engineered hydrogels (Lam et al., 2014; Ha et al., 2015; Park et al., 2017; Li et al., 2018; Lu et al., 2018; Jia et al., 2019; Pascual-Garrido et al., 2019; Wang et al., 2019; Xu et al., 2019; Cho et al., 2020), engineered nanofiber products (Shimomura et al., 2014; Li et al., 2015a; Dashtdar et al., 2015; Ruan et al., 2017; Jin et al., 2019; Kondo et al., 2019; Wu et al., 2020; Deng et al., 2021; Murata et al., 2022), and biological products (Goodrich et al., 2016; Muttigi et al., 2018; Ye et al., 2018; Park et al., 2019; You et al., 2020; Heirani-Tabasi et al., 2021; Zheng et al., 2022) (Figure 1). For details regarding structural outcomes following treatment delivery in both small and large animal OCD models, readers are directed to recent reviews by Jiang (Jiang et al., 2021) and Liu (Liu et al., 2022). In a narrative review, Jiang outlined that the current biomaterials and scaffolds used for the delivery of stem cells still have barriers to overcome, including the biocompatibility and degradation effects of polymer materials, as well as a need for better understanding regarding the effect of the degrading scaffold on stem cell activity and functionality in the body (Jiang et al., 2021). Though the majority of studies included in the review demonstrated improvement in structural outcomes, screening for the most regenerative subgroups of stem cells and preventing premature stem cell differentiation were also recommended (Jiang et al., 2021). Liu performed a narrative review summarizing MSC therapies for articular cartilage regeneration in large animals (Liu et al., 2022). Liu concluded that, given the biomechanical/anatomical differences and potent healing capacity of small animals, preclinical studies should utilize large animal models to adequately develop translatable therapeutics to humans, and that there was no clear consensus or standard regarding critical aspects of MSC therapy (e.g., cell concentration at implantation, need for pre-differentiation) for large animal cartilage regeneration (Liu et al., 2022).

## 3 Animal models of osteoarthritis

Small animal models of OA have the advantage of allowing larger sample size, less animal housing space requirement, and reduced cost *versus* larger animal models (Mukherjee et al., 2022). Small animal models of OA include mouse, rat, guinea pig and rabbit (Sato et al., 2012; Toghraie et al., 2012; ter Huurne et al., 2012; van Buul et al., 2014; Kim et al., 2014; Singh et al., 2014; Deng et al., 2015; Kuroda et al., 2015; Saulnier et al., 2015; Wang et al., 2015; Yang et al., 2015; Chiang et al., 2016; Hermeto et al., 2016; Latief et al., 2016; Li et al., 2016; Morille et al., 2016; Ozeki et al., 2016; Mei et al., 2017a; Mei et al., 2017b; Parrilli et al., 2017; Chang et al., 2018; Choi et al., 2018; Desando et al., 2018; Fan et al., 2018; Neybecker et al., 2018; Cheng et al., 2019; Desando et al., 2019; Mahmoud et al., 2019; McKinney et al., 2019; Vinod et al., 2019; Zhou et al., 2019; Jeon et al., 2020; Tong et al., 2020; Wang et al., 2020; Xing et al., 2020; Zhi et al., 2020; Tang et al., 2021; Xing et al., 2021; Juskovic et al., 2022; Kwapisz et al., 2022; Wang et al., 2023) (Table 2). Techniques to initiate OA include anterior cruciate transection (ACLT), ACLT with destabilization of the medial meniscus (DMM), chemically- (e.g., Mono-iodoacetate [MIA] or papain intraarticular injection) or collagenase-induced, as well as spontaneous OA (Culley et al., 2015). The end-result of these OA-initiating techniques is to recapitulate the total joint involvement of human OA (Culley et al., 2015; Cope et al., 2019). Contrary to OCD, the entire articular surface may be involved, and must be considered when initiating a treatment. In order to treat the entire articular surface, investigators have largely relied on fluid-based vehicles to deliver their regenerative biological elements, including saline/PBS, serum, and media (Toghraie et al., 2012; ter Huurne et al., 2012; van Buul et al., 2014; Singh et al., 2014; Saulnier et al., 2015; Yang et al., 2015; Latief et al., 2016; Li et al., 2016; Ozeki et al., 2016; Mei et al., 2017b; Chang et al., 2018; Choi et al., 2018; Fan et al., 2018; Neybecker et al., 2018; Cheng et al., 2019; Desando et al., 2019; Mahmoud et al., 2019; Zhou et al., 2019; Jeon et al., 2020; Tong et al., 2020; Zhi et al., 2020; Tang et al., 2021; Xing et al., 2021; Juskovic et al., 2022; Wang et al., 2023) (Table 2; Figure 1). More recently, HA has become commonly used (Sato et al., 2012; Deng et al., 2015; Kuroda et al., 2015; Wang et al., 2015; Chiang et al., 2016; Vinod et al., 2019; Wang et al., 2020; Xing et al., 2020; Kwapisz et al., 2022). As well, semi-permeable microspheres, such as Poly (D,L-lactide-co-glycolide)-poloxamer P188-poly (D,L-lactideco-glycolide) polymer microspheres (Morille et al., 2016) and sodium alginate microspheres (McKinney et al., 2019), have been used to encapsulate bone marrow-derived MSCs. The delivery of these encapsulated MSCs seeks to capitalize on their anabolic paracrine activity such as facilitation of tissue remodeling, recruitment of stem and progenitor cells and modulation of the immune response through the secretion of factors (e.g., interleukin-6 and stromal-cell-derived factor-1) (Morille et al., 2016; McKinney et al., 2019). Studies evaluating vehicles for both direct engraftment of stem cells, as well as paracrine-emphasizing, reported positive structural chondrogenic outcomes (Morille et al., 2016; McKinney et al., 2019; Vinod et al., 2019; Wang et al., 2020; Xing et al., 2020).

**TABLE 2:** Small animal models of OA and associated injection vehicle.

Author (Year)	Animal (n)	OA model	Joint injected	Cell type	Vehicle
Sato et al. (2012)	Guinea pig (60)	Spontaneous OA	Knee	BM-MSCs	PBS or HA
Toghraie et al. (2012)	Rabbit (20)	ACLT	Knee	AT-MSCs	Culture media
ter Huurne et al. (2012)	Mouse (n/a)	Collagenase	Knee	AT-MSCs	Mouse serum
van Buul et al. (2014)	Rat (24)	MIA	Knee	BM-MSCs	Saline
Kim et al. (2014)	Rat (38)	ACLT + MCLT + MX	Knee	BM-MSCs	Self-assembled peptide hydrogels
Singh et al. (2014)	Rabbit (20)	ACLT	Knee	BM-MSCs	Culture media
Deng et al. (2015)	Rat (28)	Papain solution and cysteine	Knee	PB-MSCs	HA
Kuroda et al. (2015)	Rabbit (12)	ACLT	Knee	AT-MSCs	HA
Saulnier et al. (2015)	Rabbit (30)	MX	Knee	Wharton’s jelly UC-MSCs	PBS
Wang et al. (2015)	Rabbit (12)	ACLT + MX	Knee	AT-MSCs	HA
Yang et al. (2015)	Rat (40)	ACLT + MX	Knee	BM-MSCs	PBS
Chiang et al. (2016)	Rabbit (28)	ACLT	Knee	BM-MSCs	HA
Hermeto et al. (2016)	Rabbit (24)	Collagenase	Knee	AT-MSCs	PRP
Latief et al. (2016)	Rat (48)	ACLT + MX	Knee	AT-MSCs	PBS
Li et al. (2016)	Rat (18)	MX		AT-MSCs	PBS
Morille et al. (2016)	Mouse (n/a)	Collagenase	Knee	BM-MSCs	Poly (D,L-lactide-co-glycolide)-poloxamer P188-poly (D,L-lactideco-glycolide) polymer microspheres
Ozeki et al. (2016)	Rat (76)	ACLT	Knee	SD-MSCs	PBS
Mei et al., (2017a)	Rat (60)	ACLT	Knee	AT-MSCs	PBS or Xanthan gum
Mei et al. (2017b)	Rat (66)	ACLT	Knee	AT-MSCs	PBS
Parrilli et al. (2017)	Rabbit (12)	ACLT	Knee	AT-MSCs	4% rabbit serum albumin
Chang et al. (2018)	Mouse (18)	MIA	Knee	UC-MSCs	Saline
Choi et al. (2018)	Rabbit (15)	ACLT		AT-MSCs	Saline
Desando et al. (2018)	Rabbit (36)	ACLT	Knee	BM-MSCs	“Physiological solution” or HA
Fan et al. (2018)	Rat (30)	MX	Knee	Pre-cartilaginous (cartilage-derived) stem cells	PBS
Neybecker et al. (2018)	Rat (32)	ACLT	Knee	SD-MSCs	Saline
Cheng et al. (2019)	Rat (30)	ACLT + MX	Knee	Wharton’s jelly MSCs	PBS
Desando et al. (2019)	Rabbit (18)	ACLT	Knee	AT-MSCs	“Physiological solution”
Mahmoud et al. (2019)	Rabbit (15)	ACLT	Knee	BM-MSCs	PBS
McKinney et al. (2019)	Rat (39)	MCLT + MX	Knee	BM-MSCs	Hanks balanced salt solution and sodium alginate microspheres
Vinod et al. (2019)	Rabbit (13)	MIA	Knee	Chondroprogenitor cells	HA
Zhou et al. (2019)	Rat (not reported)	ACLT + MCLT + MX	Knee	AT-MSCs	PBS
Jeon et al. (2020)	Rabbit (23)	ACLT	Knee	UC-MSCs	Saline
Tong et al. (2020)	Rat (n/a)	MIA	Knee	UC-MSCs	Saline
Wang et al. (2020)	Rat (n/a)	MIA	Knee	Placental MSCs	HA
Xing et al. (2020)	Rat (18)	ACLT + MX	Knee	UC-MSCs	HA
Zhi et al. (2020)	Rat (n/a)	MCLT	Knee	BM-MSCs	DMEM
Tang et al. (2021)	Rat (24)	ACLT	Knee	UC-MSCs or UC-MSC-EVs	PBS
Xing et al. (2021)	Rat (28)	ACLT	Knee	UC-MSCs	PBS
Juskovic et al. (2022)	Rat (32)	MIA	Knee	AT-MSCs	Saline
Kwapisz et al. (2022)	Guinea pig (24)	Spontaneous	Knee	AT-MSCs or Placental MSCs	HA
Wang et al. (2023)	Rat (32)	ACLT + MCLT + MX	Knee	UC-MSCs	Saline

ACLT, anterior cruciate ligament transection; AT, adipose tissue-derived; BM, bone marrow derived; DMEM, Dulbecco’s modified eagle medium; EV, extracellular vesicles; HA, hyaluronic acid; MIA, monoiodoacetate; MX, medial meniscectomy; MSCs, mesenchymal stromal cells; PBS, phosphate buffered saline; PRP, platelet rich plasma.

Large animal OA models have the advantage of more closely approximating human joints (e.g., with respect to cartilage thickness, articular biomechanics) and can more closely recapitulate OA to the human condition (Mukherjee et al., 2022). Large animal model OA studies (Al Faqeh et al., 2012; Guercio et al., 2012; Jiang et al., 2014; Song et al., 2014; Tsai et al., 2014; Ude et al., 2014; Delling et al., 2015; Desando et al., 2016; Yun et al., 2016; Barrachina et al., 2018; Feng et al., 2018; Lv et al., 2018; Srzentić Dražilov et al., 2018; Ude et al., 2018; Cabon et al., 2019; Jiang et al., 2019; Oh et al., 2021; Sanghani-Kerai et al., 2021; Armitage et al., 2022) include the use of dog, sheep, monkey and horse (Table 3). Like the small animal OA models, large animal models include surgically- (DMM, ACLT) and chemically-induced (MIA or papain intra-articular injection) methods. Similar to small animal, stem cell delivery for large animal OA studies over the last decade have largely relied on fluid-based vehicles including saline or culture media (Al Faqeh et al., 2012; Jiang et al., 2014; Song et al., 2014; Tsai et al., 2014; Ude et al., 2014; Delling et al., 2015; Barrachina et al., 2018; Srzentić Dražilov et al., 2018; Ude et al., 2018; Cabon et al., 2019; Jiang et al., 2019; Oh et al., 2021), and biological products such as HA or platelet-rich plasma (Guercio et al., 2012; Desando et al., 2016; Yun et al., 2016; Feng et al., 2018; Lv et al., 2018; Sanghani-Kerai et al., 2021; Armitage et al., 2022) (PRP, Table 3 and Figure 1). Outcomes across the majority of studies included inhibited cartilage degeneration (Morille et al., 2016; Chang et al., 2018), increased collagen II expression (Chang et al., 2018), reduced inflammatory markers (Cheng et al., 2019; Zhou et al., 2019; Wang et al., 2020) and reduced catabolic markers (Desando et al., 2016; Cheng et al., 2019). A detailed description of the outcomes reported in many of these small and large animal studies is well-summarized in by Wang (Wang et al., 2022) and Liu (Liu et al., 2022). Wang performed a systematic review in knee osteoarthritis (OA) that included 72 preclinical studies, focusing on the characteristics of animal models and cell doses, using MSCs for the treatment of knee OA (Wang et al., 2022). Wang found a correlation between the weight of the animals used and the dose of MSCs applied in the experimental treatment; however, while cartilage recovery was reported in the majority of studies, there did not seem to be a dose-response relationship across the preclinical studies included in the review (Wang et al., 2022).

**TABLE 3:** Large animal models of OA and associated injection vehicle.

Author (Year)	Animal (n)	OA model	Joint injected	Cell type	Vehicle
Al Faqeh et al. (2012)	Sheep (16)	ACLT + MX	Knee	BM-MSCs	Culture media
Guercio et al. (2012)	Dog (4)	Spontaneous OA	Knee	AT-MSCs	PRP or HA
Jiang et al. (2014)	Rhesus macaques (27)	Collagenase	Knee	BM-MSCs	Normal saline
Song et al. (2014)	Sheep (18)	ACLT + MX	Knee	BM-MSCs	PBS
Tsai et al. (2014)	Dog (3)	Spontaneous OA	Knee	AT-MSCs	PBS
Ude et al. (2014)	Sheep (18)	ACLT + MX	Knee	Chondrogenically-induced AT-MSCs and BM-MSCs	Culture media
Delling et al. (2015)	Sheep (12)	MX	Knee	BM-MSCs	PBS
Desando et al. (2016)	Sheep (20)	MX	Knee	BM-MSCs	HA
Yun et al. (2016)	Dog (24)	ACLT	Knee	AT-MSCs	PBS or PRP
Barrachina et al. (2018)	Horse (18)	Amphotericin-B	Knee	BM-MSCs	RL
Feng et al. (2018)	Sheep (30)	ACLT + MX	Knee	AT-MSCs	HA
Lv et al. (2018)	Sheep (34)	ACLT + MX	Knee	AT-MSCs	HA
Srzentić Dražilov et al. (2018)	Dog (10)	Spontaneous OA	Various	AT-MSCs	PBS
Ude et al. (2018)	Sheep (18)	ACLT + MX	Knee	Chondrogenically-induced AT-MSCs and BM-MSCs	Culture media
Cabon et al. (2019)	Dog (22)	Spontaneous OA	Various	MSCs from neonatal tissues	PBS
Jiang et al. (2019)	Rhesus macaques (8)	Spontaneous OA	Knee	Embryonic or BM-MSCs	Normal saline
Oh et al. (2021)	Dog (16)	ACLT	Knee	AT-MSCs	Normal saline
Sanghani-Kerai et al. (2021)	Dog (25)	Spontaneous OA	Various	AT-MSCs	PRP
Armitage et al. (2022)	Dog (245)	Spontaneous OA	Various	AT-MSCs	PRP

ACLT, anterior cruciate ligament transection; AT, adipose tissue-derived; BM, bone marrow derived; HA, hyaluronic acid; MSCs, mesenchymal stromal cells; MX, meniscectomy; PBS, phosphate buffered saline; PRP, platelet rich plasma; RL, ringer’s lactate.

It is critical to note that neither small nor large OA animal models used the more advanced tissue engineering stem cell delivery methods (Figure 1). This is likely owing to the diffuse nature of articular cartilage loss in the OA joint, making it difficult to engineer or “print” a scaffold that could be applied the non-uniform and diffusely compromised articular surface.

## 4 Clinical studies of focal cartilage injury and osteoarthritis

Clinical studies on focal cartilage defects (Buda et al., 2013; Saw et al., 2013; Akgun et al., 2015; Koh et al., 2016; de Windt et al., 2017; Kamei et al., 2018; Shimomura et al., 2018; de Girolamo et al., 2019; Gobbi and Whyte, 2019; Hashimoto et al., 2019; Monckeberg et al., 2019; Pipino et al., 2019; Kyriakidis et al., 2020; Neckar et al., 2022; Shetty et al., 2022; Venosa et al., 2022; Abdelhamid et al., 2023; Song et al., 2023) (Table 4) often included individuals with isolated cartilage lesions of International Cartilage Repair Society (ICRS) grades 3–4 or equivalent (severely abnormal cartilage with loss of ≥50% cartilage depth). Vehicles included liquid delivery systems such as saline (Kamei et al., 2018; Shimomura et al., 2018), HA (Buda et al., 2013; Saw et al., 2013; Hashimoto et al., 2019; Shetty et al., 2022; Song et al., 2023), and PRP (Monckeberg et al., 2019; Venosa et al., 2022). Scaffolds included HA- or collagen-based membranes (Buda et al., 2013; Akgun et al., 2015), fibrin glue (Koh et al., 2016; de Windt et al., 2017; de Girolamo et al., 2019), or tissue-engineered materials (Gobbi and Whyte, 2019; Pipino et al., 2019; Kyriakidis et al., 2020; Neckar et al., 2022; Abdelhamid et al., 2023) (Figure 1). Fluid-based vehicles were often injected under arthroscopic guidance, while scaffolds shaped to match the focal cartilage defect were often placed using mini arthrotomy (Table 4). As with the OA clinical trials, the vast majority of the studies reported that the interventions led to positive clinical outcomes. Many of these studies reported improved cartilage structural outcomes assessed by MRI. Summary of these outcomes for clinical OCD studies are presented by Jiang (Jiang et al., 2021) and Lv (Lv et al., 2023). In a narrative review, Lv concluded that while MSCs have therapeutic value, a stronger evidence base is needed until they can be clinically applied (Lv et al., 2023). It was also noted that injecting MSCs has significant limitations, including cell death at injection and significant leakage at the injection site and that overcoming these limitations via biomaterial-assisted cell cartilage repair may be the optimal direction for OA treatment (Lv et al., 2023).

**TABLE 4:** Clinical studies using stem cells for isolated chondral lesions and associated injection vehicle.

Author (Year)	Chondral defect	Joint (n)	Cell type	Vehicle	Implantation method
Buda et al. (2013)	ICRS grade 3–4	Knee (30)	BM-MSCs	HA membrane or collagen membrane	Arthroscopic placement
Saw et al. (2013)	ICRS grade 3–4	Knee (50)	PB-MSCs	HA	Post-op injection
Akgun et al. (2015)	Single full-thickness femoral condylar chondral defects	Knee (18)	SD-MSCs	Collagen I/III membrane shaped to lesion and sutured in place	Open mini-arthrotomy
Koh et al. (2016)	Single symptomatic cartilage lesion ≥3 cm^2^	Knee (80)	AT-MSCs	Fibrin glue (cell-thrombin suspension + fibrinogen)	Injection under arthroscopic guidance
de Windt et al. (2017)	full-thickness defect of 2–8 cm^2^	Knee (35)	BM-MSCs	Fibrin glue with chondrocytes	Open mini-arthrotomy
Kamei et al. (2018)	>50% thickness, >2 cm^2^	Knee (5)	BM-MSCs	Saline	Injection under arthroscopic guidance
Shimomura et al. (2018)	ICRS grade 3–4	Knee (5)	SD-MSCs	MSCs and their ECM in saline	Arthroscopic placement
de Girolamo et al. (2019)	ICRS grade 3–4, 2–8 cm^2^	Knee (24)	BM-MSCs	Chondro-Gide^®^ collagen type I/III bilayer matrix and fibrin glue	Open mini-arthrotomy
Gobbi and Whyte (2019)	ICRS grade 4, ≥1 cm^2^	Knee (23)	BM-MSCs	Hyalofast^®^ non-woven benzyl ester of HA biodegradable scaffold	Open mini-arthrotomy
Hashimoto et al. (2019)	ICRS grade ≥3, ≥2 cm^2^	Knee (11)	BM-MSCs	HA	Injection under arthroscopic guidance
Monckeberg et al. (2019)	ICRS grade 3b	Knee (20)	PB-MSCs	PRP	Injection under arthroscopic guidance
Pipino et al. (2019)	Outerbridge III–IV	Knee (69)	AT-MSCs	JointRep™ PG/GC hydrogel matrix	Arthroscopic placement
Kyriakidis et al. (2020)	ICRS grade 3–4, >2 cm^2^	Knee (25)	AT-MSCs	Hyalofast^®^ non-woven benzyl ester of HA biodegradable scaffold	Arthroscopic placement
Neckar et al. (2022)	Outerbridge III–IV, 1.9 ± 0.3 cm^2^	Knee (6)	BM-MSCs	Chondrotissue^®^ resorbable scaffold composed of PA and HA, Tisseel fibrin sealant	Open mini-arthrotomy
Shetty et al. (2022)	ICRS grade 3–4, ≤8 cm^2^	Knee (32)	BM-MSCs	HA	Post-op injection
Venosa et al. (2022)	Outerbridge IV < 2 cm^2^	Knee (38)	AT-MSCs	PRP	Injection under arthroscopic guidance
Abdelhamid et al. (2023)	Single focal, full-thickness cartilage defect 2–10 cm^2^<	Knee (10)	AT-MSCs with allogenic hyaline cartilage powder	Solidified autograft with allogenic hyaline cartilage powder implants on a 3D printed polycaprolactone scaffold, with fibrin glue	Open limited arthrotomy
Song et al. (2023)	Outerbridge III–IV, >4 cm^2^	Knee (85)	UC-MSCs	Sodium hyaluronate	Open mini-arthrotomy

AT, adipose tissue-derived; BM, bone marrow derived; HA, hyaluronic acid; ICRS, international cartilage repair society; MSCs, mesenchymal stromal cells; PB, peripheral blood; PBS, phosphate buffered saline; PA, polyglycolic acid; PG/GC, polyglucosamine/glucosamine carbonate; PRP, platelet rich plasma; SD, synovial-derived; UC, umbilical cord-derived.

Like small and large animal models of OA, randomized controlled trials evaluating stem use for the treatment of OA in humans (Vega et al., 2015; Gupta et al., 2016; Lamo-Espinosa et al., 2016; Goncars et al., 2017; Shapiro et al., 2017; Emadedin et al., 2018; Freitag et al., 2019; Lee et al., 2019; Lu et al., 2019; Matas et al., 2019; Anz et al., 2020; Bastos et al., 2020; Garza et al., 2020; Lamo-Espinosa et al., 2020; Chen et al., 2021) included a broad spectrum of OA severity (KL grades 1–4 included) and have largely made use of fluid-based vehicles such as saline (Goncars et al., 2017; Emadedin et al., 2018; Freitag et al., 2019; Lee et al., 2019; Matas et al., 2019), Ringer’s lactate (Vega et al., 2015; Garza et al., 2020), proprietary electrolyte solutions (Gupta et al., 2016; Lu et al., 2019; Chen et al., 2021), HA (Lamo-Espinosa et al., 2016), or PRP (Anz et al., 2020; Bastos et al., 2020; Lamo-Espinosa et al., 2020) (Table 5). Stem cell delivery was often performed by intra-articular injection, with or without imaging guidance. These studies generally reported no major adverse events with the treatment provided. A majority of trials reported improved clinical outcomes, though no study reported improved structural outcomes. Clinical outcomes reported by these trials were summarized by Jiang (Jiang et al., 2021) and Lv (Lv et al., 2023) in the reviews noted above.

**TABLE 5:** Human randomized controlled trials using stem cells for knee OA and associated injection vehicle.

Author (Year)	OA severity (n)	Cell type	Vehicle	Injection guidance method
Vega et al. (2015)	KL 2–4 (30)	BM-MSCs	RL + glucose	Anatomic
Gupta et al. (2016)	KL 2–3 (60)	BM-MSCs	PLASMA-LYTE (electrolyte solution)	Anatomic
Lamo-Espinosa et al. (2016)	KL ≥ 2 (30)	BM-MSCs	HA	Anatomic
Goncars et al. (2017)	KL 2–3 (56)	BM-MSCs	Saline	Anatomic
Shapiro et al. (2017)	KL 1–3 (50)	BM-MSCs	Platelet-poor bone marrow plasma	U/S
Emadedin et al. (2018)	KL 2–4 (47)	BM-MSCs	Saline	X-ray
Freitag et al. (2019)	KL 2–3 (30)	AT-MSCs	Saline	U/S
Lee et al. (2019)	KL 2–4 (24)	AT-MSCs	Saline	U/S
Lu et al. (2019)	KL 1–3 (53)	AT-MSCs	Re-join^®^ cell suspension solution (proprietary)	U/S
Matas et al. (2019)	KL 1–3 (29)	UC-MSCs	Saline + plasma	Anatomic
Anz et al. (2020)	KL 1–3 (90)	BM-MSCs	Plasma	U/S
Bastos et al. (2020)	KL 1–4 (47)	BM-MSCs	PBS or PRP	Anatomic
Garza et al. (2020)	KL 2–3 (39)	AT-MSCs	RL	U/S
Lamo-Espinosa et al. (2020)	KL ≥ 2 (60)	BM-MSCs	PRP	Anatomic
Chen et al., (2021)	KL 1–3 (57)	AT-MSCs	ELIXCYTE^®^ cell suspension solution (proprietary)	Anatomic

AT, adipose tissue-derived; BM, bone marrow derived; HA, hyaluronic acid; KL, Kellgren and Lawrence radiographic OA grade; MSCs, mesenchymal stromal cells; PBS, phosphate buffered saline; PRP, platelet rich plasma; RL, ringer’s lactate; UC, umbilical cord-derived.

## 5 Lessons learned from the current literature

Emerging technologies, including 3D printing of biological scaffolds, allow the standardized computer-assisted creation of delivery vehicles with precise control over composition, pore size, stiffness and geometry, reviewed in detail by Kim (2019) (Kim et al., 2019) and Lafuente-Merchan (2022) (Lafuente-Merchan et al., 2022). These differing 3D environments influence migration, cell-cell integrin interactions, signaling and phenotype of the cells encapsulated within the scaffold (Kim et al., 2019; Lafuente-Merchan et al., 2022). These regenerative cells, as well as growth factors important for regenerative cell viability, proliferation and differentiation can be embedded in the scaffold, adapted specifically to the tissue requiring repair (Kim et al., 2019; Campbell, 2020). Solubility of the scaffold can be adjusted to emphasize anabolic paracrine functionality of incorporated cells as well as growth factor release rate (Murata et al., 2022). Overall, it is anticipated that these combined advantages will provide superior cartilage regeneration capacity for injured cartilage (Mandrycky et al., 2016; Kim et al., 2019; Lafuente-Merchan et al., 2022).

Ultimately, the ideal regenerative vehicle could treat all forms of cartilage injury, including OCDs and OA. The development of such vehicles was unfortunately delayed by the clinical use of stem cells for cartilage regeneration prior to establishing a strong evidence base (Marks et al., 2017; Canada, 2019; ISfSC, 2021). Formal regulation of stem cell application in the clinical setting created considerable movement forward towards this goal of applying tissue engineering technology to cartilage regeneration. Based on the available literature, advanced multi-layered scaffolds with molecular and cellular composition gradients optimized for the tissues to be repaired are applied to animal models of OCD (Wu et al., 2020; Deng et al., 2021; Murata et al., 2022). Implantation of nanopatterned differentiated MSCs as stratified bilayered hydrogel constructs improved the repair quality of cartilage defects, as indicated by histological scoring, mechanical properties, and polarized microscopy analysis in rabbit (Wu et al., 2020). A GM + SF-PTH/GM + SF-MA osteochondral biphasic scaffold promoted the regeneration of OCDs in rabbit and maintained hyaline cartilage phenotype, as evaluated by histologic staining and immunohistochemistry (Deng et al., 2021). Synovial fluid-derived MSCs contained within 3D spheroids reduced the sizes of OCDs in an equine model as seen on MRI and the histology-based ICRS scale (Murata et al., 2022). This technology has been piloted in clinical studies of OCD (Abdelhamid et al., 2023), with positive structural results (Jiang et al., 2021; Lv et al., 2023). Given the large number of individuals affected by OCD, the research advances represent an important step forward (Mandelbaum et al., 1998; Ding et al., 2007; Everhart et al., 2019; Liu et al., 2022). Presently, however applying these technologies to OA, the most common musculoskeletal disease worldwide, faces more challenges (Barr et al., 2015; Martel-Pelletier et al., 2016; Hunter and Bierma-Zeinstra, 2019; Campbell and McGonagle, 2020). One of the challenges presented by the OA joint is the heterogeneous quality of diffuse articular cartilage damage. Studies treating OCD have the advantage of “filling” a lesion of pre-defined dimensions (in the case of animal models), or that is limited in its dimensions (in the case of clinical OCDs). In OA, the articular damage is often less delineated, making it difficult to pre-fabricate a scaffold that will treat the entire articular surface. An analogy would be the repair of a pothole (OCD) *versus* repaving the entire road (OA). Other challenges in OA regenerative treatment includes the non-chondrogenic contributors to the OA joint (e.g., subchondral bone changes, synovial hypertrophy, altered forces across the joint due to abnormal joint alignment, etc.) that must also be considered (McGonagle et al., 2017).

## 6 Future directions

Scaffold engineering advances for OCD are progressing rapidly with ever-more sophisticated methods of delivery. For those with OA, current strategies to optimize the delivery vehicle have included the use of existing biological fluids such as PRP, or synthesized biological material such as HA. PRP has been cited to reduce the occurrence of inflammation, improve angiogenesis, and promote the proliferation and differentiation of chondrocytes by secreting cytokines, chemokines, and growth factors, as well as exosomes with similar effects, so as to promote the healing of bone and cartilage injuries (Liang et al., 2022). In clinical studies, patients with knee OA treated with PRP showed improvement in physical function and pain, suggesting that intra-articular injection of PRP may be a potential therapeutic strategy for relieving knee pain (Liang et al., 2022; Riewruja et al., 2022). HA is a naturally-occurring, non-toxic, biodegradable biopolymer essential for bone growth and chondrocyte differentiation, and has been reported to stimulate stem cell chondrogenesis as well as cartilage-specific ECM production by stem cells (Matsumoto et al., 2009; Yoon et al., 2011; Toh et al., 2012; Davoudi et al., 2018; Park et al., 2020). HA may also provide a protective milieu for the cells in the damaged joint surface environment and ensure cells remain localized to cartilage injury (Li et al., 2015b; Campbell, 2020). HA is used clinically to treat OA via intra-articular application (Chavda et al., 2022). For OA, vehicles such as PRP and HA may represent a compromise between form and function, with custom and commercial versions of each being developed towards regenerative optimization (Gato-Calvo et al., 2019; Chavda et al., 2022).

Ideally, tissue engineering advances for cartilage repair will 1 day be applicable to the OA joint. Possible solutions for matching OA cartilage loss may include joint surface modeling using CT or MRI that would allow the printing of a cartilage scaffold to be overlain on the damaged articular surface. Such an approach is currently being evaluated for the custom manufacturing of total knee arthroplasty components (Beckmann et al., 2020; Moret et al., 2021), but might also be applicable to cartilage regeneration. Applying a fluidic vehicle that will polymerize over the articular surface at body temperature or with another catalyzing stimulus, such as specific wavelengths of light, could secure a regenerating agent in place while permitting the supportive benefits of the scaffold. Concurrent treatment, such as joint distraction or alignment correction (McGonagle et al., 2017; Jansen and Mastbergen, 2022) may be of benefit when applying cartilage regeneration treatments to the OA joint in order to reduce the potential for damage to the treatment components during the regeneration process.

## 7 Conclusion

Regenerative stem cell delivery for cartilage repair has advanced considerably over the last decade with the most impressive advances incorporating both structural and biological features designed to enhance cartilage repair. At present these advances are being directed towards focal OCDs in animal models, foreshadowing competition with popular biological products such as HA and PRP. While the application of such technologies to OCD in the clinical setting is likely just around the corner, whether their application can be adapted to the more diffusely damaged articular surface of the common and debilitating OA joint is yet to be seen, and whether they prove superior to biological agents such as HA or PRP in the OA setting is yet to be demonstrated.
